# Multiplex optical detection and quantification of DNA fragments by metallo-peptide assemblies

**DOI:** 10.1038/s41598-019-45124-z

**Published:** 2019-06-19

**Authors:** Abhijit Saha, Meital Reches

**Affiliations:** 1 Institute of Chemistry, The Hebrew University of Jerusalem, Edmond J. Safra Campus, Givat Ram, Jerusalem, 9190401 Israel; 2 The center for Nanoscience and Nanotechnology, The Hebrew University of Jerusalem, Jerusalem, 9190401 Israel

**Keywords:** Materials chemistry, Nanoscale materials

## Abstract

Rapid detection of infectious agents such as bacteria and viruses are important for proper health management, agriculture and homeland security. This paper presents a multiplex DNA detection system self-assembled by a metallo-peptide complex. Within five minutes, the system can simultaneously detect multiple DNA fragments, without any need for their separation. The presence of proteins in the sample does not harm the detection capabilities of the system, which can discriminate even between one base-pair mismatch and can perform at concentrations as low as 200 pM.

## Introduction

Rapid detection of infectious threats is a crucial first step for good management of health and bioterrorism^1^. Early detection of cancer and identification of the tumor mutation is very important for providing the best treatment for cancer patients. With bacterial or viral infections caused by anthrax or bacillus anthracis, for example, the pathogenic agent quickly multiplies and spreads. It can subsequently kill the patient if a specific, needed treatment is not provided within 48 hours. Therefore, rapid detection and quantification of several DNA fragments simultaneously is important for medical diagnosis, pathology, molecular biology research, biomedical studies and mutation analysis^2–5^. The traditional culture-based laboratory method for detecting pathogenic DNA is time consuming and requires 24–72 hours. In addition, administering antibiotics prior to sample collection inhibits the positive identification of pathogenic DNA. Many techniques have been developed, based on DNA hybridization because of its high selectivity and specificity, which overcome the problems associated with culture-based methods^6–8^. Recent research has developed a variety of electrochemical and optical DNA hybridization sensors. Some examples include DNA micro array analysis^9^, use of quantum dots and semiconductor crystals as fluorescence probes^10–12^, nanoparticle-amplified surface plasma enhanced signals^13,14^ and red-ox active nucleic acid^15^. Fluorescent dye molecules are also used for signaling the DNA hybridization^16^ and there is increasing interest in developing an optical sensing platform over an electrochemical biosensor^17^. Fluorescence-based detection such as the polymerase chain reaction (PCR) currently serves as a sensitive DNA detection system that amplifies the DNA fragments^18^. However, the complex multistep process and the presence of residual DNA fragments account for the false positive results^19^. Therefore, a novel cost-effective and sensitive method is required for detecting multiple DNA fragments in a sample.

The process of self-assemble in peptide molecules leads to formation of different kind of nanostructures^20–25^ which are also able to bind with different chemical compounds and surfaces^26,27^. Our group has recently shown the use of self-assembled nanostructures generated by a metallo-peptide as an optical sensing platform for single-stranded DNA detection^28^. This system is a fluorescence-based detection method whereby a complementary DNA fragment (complementary to the desired analytic DNA) is labelled with a fluorescent dye molecule. The fluorescence of the labelled complementary DNA (termed “probe DNA”) is quenched after the addition of metal peptide conjugate (LM, ligand metal complex) and the fluorescence is recovered upon the addition of target DNA. Here, we extended the capabilities of this system and show that it can simultaneously detect not only one DNA fragment: it can also detect multiple DNA fragments in one sample. We also show that the system can perform in the presence of proteins, without pre-modification and that it incorporates a physical separation process using the metallo-peptide complex (LM). In addition, we demonstrate that the detection limit is as low as 200 pM.

## Results and Discussion

In brief, the assay is as follows. We design complementary DNA to the desired target DNA in the sample. The complementary DNA fragments are labelled at the 3′ position with a selectively chosen fluorescence dye molecule. We prepare a mixture of labelled complementary DNA fragments and LM. This mixture has very low fluorescence as the background fluorescence. This mixture is then added to the solution of target DNA and the fluorescence is recorded. The enhancement of fluorescence at the characteristic wavelengths corresponding to the respective fluorescence dye molecules indicates the presence of the suspected target DNA fragments. The synthesis, characterization of the ligand metal conjugate (LM) and the morphology of the self-assembled LM were described in our previous publication^28^. LM (Fig. S1) self-assembles into spherical aggregates at a concentration range of 2 to 3 mg/mL in water^28^. The self-assembled form of LM, having no fluorescence (Fig. S2), is used for DNA detection.

**Figure 1:**
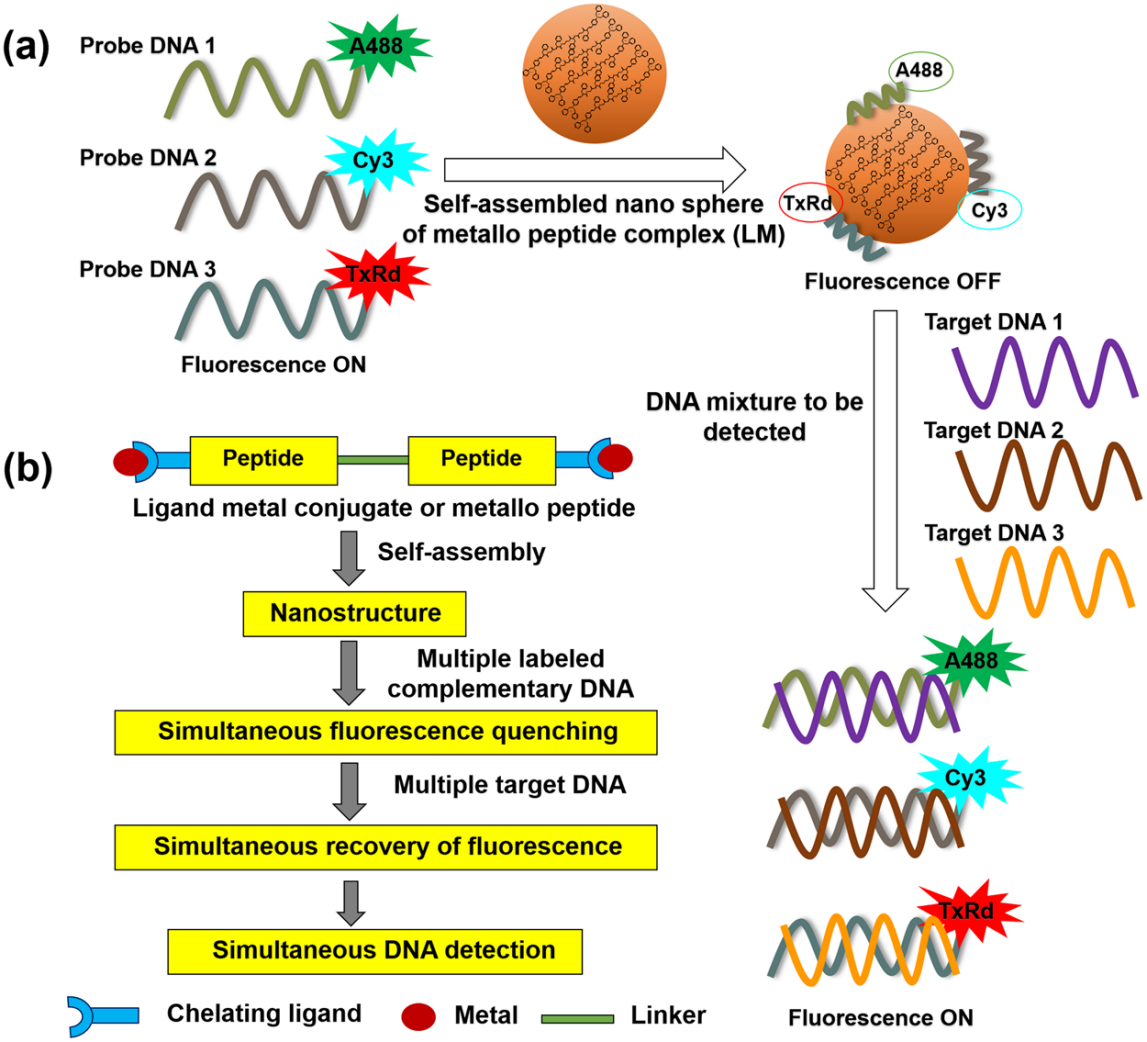
(**a**) Stepwise representation of simultaneous detection of multiple DNA fragments. Probe DNA samples are the complementary DNA corresponding to the target DNA. (**b**) Description of the detection technique.

Figure 1 illustrates the working principle of the DNA detection technique. As a proof-of-concept of multiple DNA detection, we selected three bacterial meningitis pathogens e.g., *Streptococcus pneumoniae* (DNA1), *Haemophilus influenzae* (DNA2) and *Neisseria meningitidis* (DNA3). The DNA sequences of those pathogens are presented in Table 1a (Table S1, supporting information). These constitute the target DNA. The complementary DNA corresponding to these target DNA and their 3′ modification with fluorescent dye molecules are also indicated in Table 1b (Table S1). We selected three fluorescent dye molecules, e.g., Alexa Fluor 488, Cyanine 3 and Texas Red for the 3′ modification of complementary DNA. These three dye molecules excite and fluoresce at different wavelengths. Alexa 488, Cy3 and Texas Red labelled C_DNA1, C_DNA2 and C_DNA3, respectively, are shown in Table 1b (Table S1).

**Figure 2:**
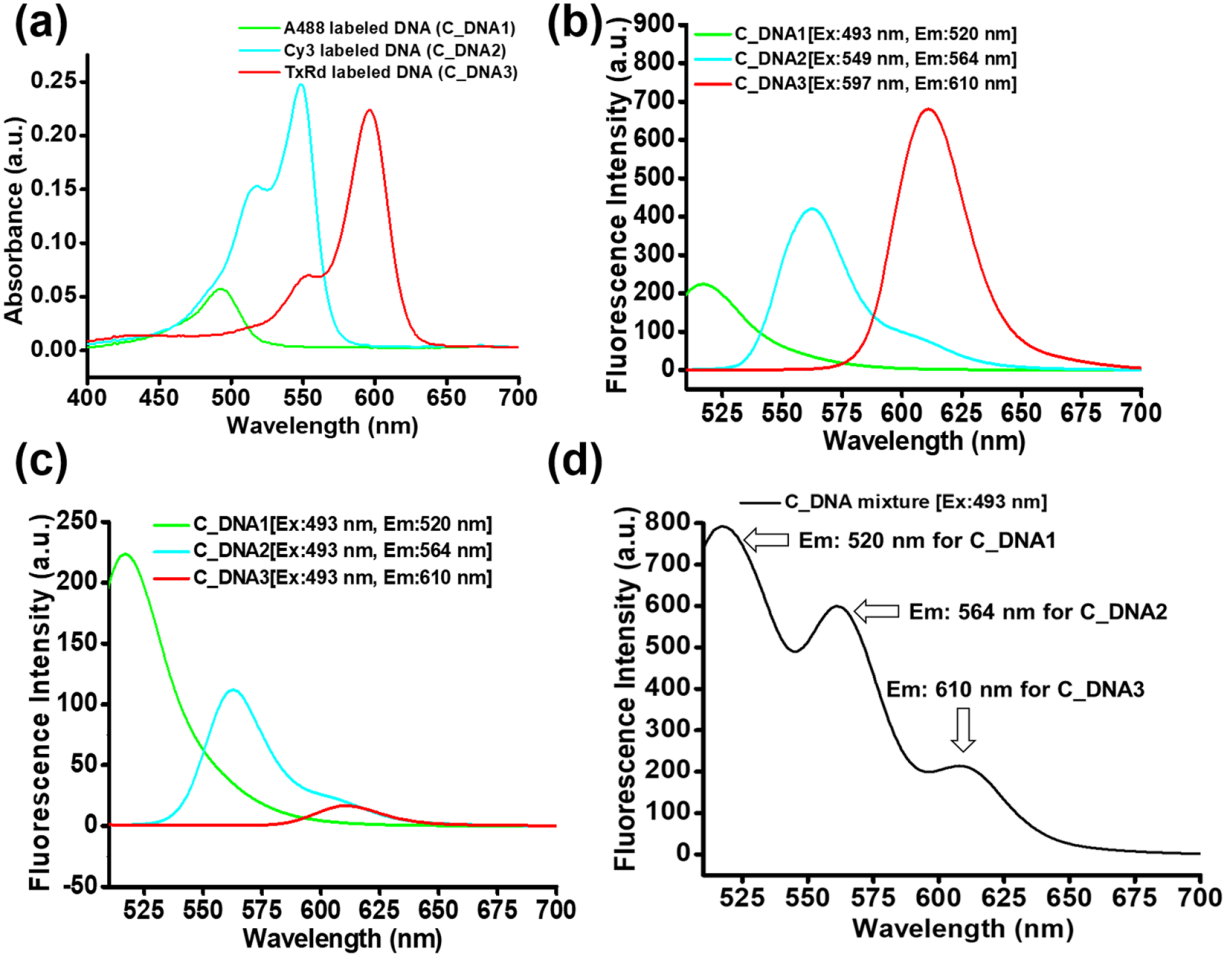
Optimization steps for detecting multiple fluorophores using a single excitation wavelength. (**a**) Individual absorbance spectra of the labelled DNA samples. (**b**) Individual fluorescence spectra of the labelled DNA samples. (**c**) Individual fluorescence spectra of the A488, Cy3 and TxRd labelled DNA samples using excitation at 493 nm. (**d**) Fluorescence spectrum of the three labelled DNA samples in a mixture using excitation at 493 nm.

**Figure 3:**
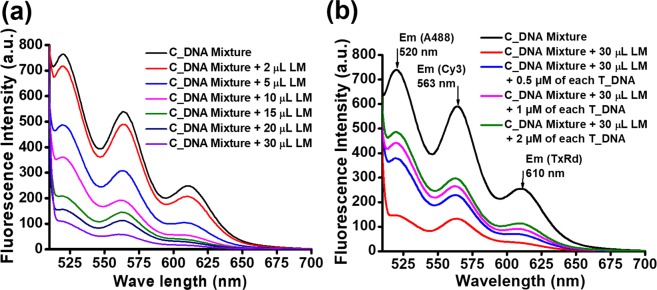
Description of simultaneous multiple DNA detection. (**a**) Quenching of the fluorescence of three probe DNA samples simultaneously with increasing amounts of LM. (**b**) Detection of three DNA samples simultaneously. Fluorescence quenching and its recovery after the addition of target DNA.

To examine the absorbance and fluorescence spectra of the labelled DNA fragments, we recorded the individual absorbance and fluorescent spectra of the labelled DNA oligomers in 0.5 mM Tris HCl buffer, pH 7.5. The individual absorbance spectra indicated maximum absorbance at 493 nm for Alexa 488, 549 nm for Cy3 and 597 nm for Texas Red labelled DNA (Fig. 2a). The individual fluorescence spectra using the excitation wavelength corresponding to the maximum absorbance showed that labelled C_DNA1, C_DNA2 and C_DNA3 fluoresce at 520 nm, 564 nm and 615 nm, respectively (Fig. 2b). Since, emission wavelengths are quite separated from each other there is less chance of overlapping. The emission spectra were recorded from 400 nm to 700 nm (Fig. S3a). The lowest wavelength used for excitation of C_DNA molecules is 493 nm. According to Jablonski diagram, the fluorescence emission occurs after the excitation wavelength. Therefore, we present the emission spectra from 510 nm to 700 nm in the main text. Each of the three C_DNA molecules is excited at a certain wavelength. However, for simultaneous excitation of the three DNA molecules a single excitation wavelength is required. Among the three excitation wavelengths (493 nm, 549 nm and 597 nm) we selected 493 nm for excitation of the three C_DNA molecules. This is because it is the lowest wavelength with the highest energy that allows the excitation of all the C_DNA molecules. We recorded the fluorescence spectra of individual C_DNA molecules (Fig. 2c) and their mixture (Fig. 2d) using excitation wavelength at 493 nm. The whole spectra from 400 nm to 700 nm were shown in supporting information (Fig. S3b,c). We observed that the fluorescence intensities of the C_DNA2 and C_DNA3 are very low when we excite them by 493 nm. Therefore, to increase the fluorescence intensity of C_DNA2 and C_DNA3, we increased their concentrations compared to C_DNA1. We optimized the concentration of the labelled complementary DNA fragments so that they could be used for multiple DNA detection. We found that a mixture of labelled probe DNA1 at 26 nM, probe DNA2 at 100 nM and probe DNA3 at 300 nM yields a well-shaped fluorescence spectrum, denoted as C-DNA mixture (Fig. 2d), shown by the black line in Fig. 3, using 493 nm as the excitation wavelength. Three different fluorescence peaks are present in the spectrum (Fig. 2d). The peak at 520 nm corresponds to the labeled C_DNA1, the peak at 563 nm corresponds to the labeled C-DNA2 and the peak at 610 nm corresponds to labeled C_DNA3. This result indicates that by using a single excitation wavelength of 493 nm we were able to excite three probe DNA fragments at the same time. We showed the individual emission spectra of those labeled C_DNA molecules (Fig. 2c) and emission spectra of the mixture (Fig. 2d). These spectra indicate that the emission wavelength of the dye molecules is similar when they are separated or in a mixture. Therefore, wavelengths overlapping was not an issue for the simultaneous detection.

Next, we self-assembled LM in water (2 to 3 mg/mL) and lyophilized it to dry the self-assembled form. The self-assembled form of LM was used for DNA detection (Supporting Information). We prepared a stock solution of 1 mg/mL of self-assembled LM in water for our study. According to our method, for simultaneous detection of multiple DNA fragments, it is important to check whether LM can quench all the fluorescence signals of the labelled probe DNA fragments simultaneously. Therefore, to study the fluorescence quenching efficacy of LM, we took the fluorescence of the mixture of labelled probe DNA fragments in the absence and presence of various amounts of LM (Fig. 3a). We found that the addition of a 2 µL solution of LM (from 1 mg/mL stock) into the labelled C_DNA mixture (mixture of labelled probe DNA fragments) slightly quenched all the fluorescence of the three labelled probe DNAs (Fig. 3a) simultaneously. A further increase in the amount of LM from 2 µL to 30 µL yielded additional quenching (Fig. 3a) simultaneously. The fluorescence quenching is instantaneous and the extent of quenching is only dependent on the amount of LM. The non-covalent π-π stacking between the aromatic moiety of LM and the nucleotide bases of the DNA molecules facilitates the interactions between the single-stranded DNA molecules to LM, leading to fluorescence quenching. The d9 electronic configuration of Cu^2+^ also assists the binding of LM to the DNA and in fluorescence quenching owing to the photo-induced electron transfer process^29^. We have observed that the mixture of labelled probe DNA fragments and 30 µL of LM exhibited a very low fluorescence signal. Thus, we have used this mixture as a reference for identification and quantification of target DNA samples.

For multiple DNA identification, we prepared a mixture containing 30 µL LM and a mixture of probe DNA fragments and added the mixture to the solution of the target DNA mixture (T_DNA), i.e., the mixture of DNA1, DNA2 and DNA3. Importantly, we observed a significant simultaneous enhancement of the fluorescence signal at 520 nm for labelled C_DNA1, at 564 nm for C_DNA2 and at 610 nm for C_DNA3 (Figs 3b and S4). The simultaneous enhancement of the fluorescence signal is concentration dependent. Therefore, we found that the fluorescence signal was gradually enhanced when the concentration of the target DNA samples was increased from 0.5 µM to 2 µM (Fig. 3b). All the fluorescence signal enhancements occurred instantly and simultaneously. Thus, the presence of multiple DNA fragments in a sample was detected by a single assay.

**Figure 4:**
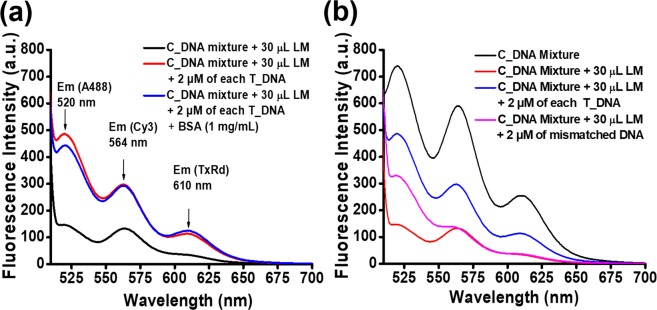
Selectivity and sensitivity of the system. (**a**) Detection of three DNA samples in the presence of BSA protein, indicating the selectivity of the system. (**b**) Distinguishing real DNA from the mismatched DNA, indicating the sensitivity of the system.

To test the sensitivity of the assay, we tried to detect multiple DNAs in the presence of proteins. To this end, we added the mixture of LM and the labelled C_DNA mixture into the 2 µM concentration of target DNA mixture (T_DNA) in the presence of bovine serum albumin (BSA) having a concentration of 1 mg/mL. We recorded the fluorescence spectra using excitation at 493 nm. Interestingly, we observed a marked enhancement of the fluorescence signal for all three DNA fragments (Fig. 4a). We also took similar fluorescence spectra in the absence of protein. We found that the enhancement of the fluorescence signal on contact with target DNA is not affected by the presence of BSA protein. We also performed similar assay in presence of skim milk (Fig. S5). These assays indicate that the system is so sensitive that it can identify multiple DNA fragments in a sample simultaneously in the presence of protein.

To examine the selectivity of our assay technique, we used mismatched DNA fragments instead of real target DNA. The DNA sequences are written in the supporting information (Table S1). In the mismatched DNA, thymine (T) replaces guanine (G). Therefore, this mismatched DNA will not hybridize completely with the corresponding complementary DNA (C_DNA) and a proper fluorescence enhancement will not occur. We recorded the fluorescence spectra of the labelled C_DNA mixture in the absence and presence of 30 µL of LM. Next, we prepared a similar mixture of LM and the labelled C_DNA mixture and added it to the 2 µM concentration of mismatched DNA, which is single base mismatched with respect to T_DNA1. As a control, we also prepared a similar mixture of LM and the labelled C_DNA mixture and added it to the solution of the real T_DNA mixture (2 µM). The fluorescence spectra were recorded using excitation at 493 nm. Interestingly, we did not observe an enhancement of the fluorescence signal for DNA2 and DNA3 when the mixture of LM and the labelled C_DNA was added to the solution containing mismatched DNA. We found a slight enhancement of the fluorescence signal for DNA1, which was much less compared with the control. This experiment indicates that our assay method is very sensitive and that it can discriminate between single base mismatched DNA fragments (Fig. 4b).

**Figure 5:**
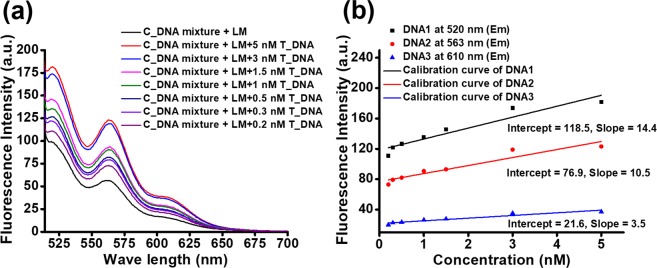
Calibration curve for simultaneous detection of three DNA samples at very low concentrations. (**a**) The enhancement of fluorescence signals decreases when the concentration of target DNA is lowered. The observed lower detection limit is 0.2 nM. (**b**) Calibration curve for detection and quantification of multiple DNA samples simultaneously.

The detection limit is important for a DNA detection assay. Thus, we were interested in determining the lower concentration of target DNA fragments in a sample that can be detected and quantified simultaneously by our novel assay technique. Therefore, we prepared different concentrations of target DNA solutions containing three DNA fragments ranging from 5 nM to 0.1 nM. We recorded the fluorescence spectra of the labelled C_DNA mixture in the presence of 30 µL of LM (Figs 5a and S6). In the figure this spectrum is denoted by a black line. Next, we prepared a similar mixture of LM and the labelled C_DNA mixture and added it to the solutions of the target DNA mixture (T_DNA). We recorded the fluorescence spectra using excitation at 493 nm.

Interestingly, we observed a marked enhancement of fluorescence signals for all three DNA fragments (Fig. 5a). However, the enhancement was decreased when we decreased the concentration of target DNA. We did not find an enhancement of the fluorescence signal for 0.1 nM target DNA; moreover, the spectrum overlaps with the spectrum of LM. Therefore, we did not show it in Fig. 5a. It is shown in Fig. S7. Regarding our observations from this experiment, we can detect multiple DNA fragments in a sample simultaneously up to a concentration of 200 pM (Fig. S6). It means that the detection is possible if the sample contains 1.2 × 10^14^ molecules of DNA. The enhancement of fluorescence at the characteristic wavelengths corresponding to the respective fluorescent dye is dependent on the concentration of the target DNA. Thus, we obtained the concentration along with a fluorescence curve for different target DNA fragments (Fig. 5b). We obtained three calibration curves for three DNA fragments at a concentration range of 0.2 nM to 5 nM. We can measure the concentration of the DNA fragments in a sample from the calibration curve. Thus, we can detect and quantify multiple DNA fragments in a sample simultaneously.

Lastly, in a control experiment we showed that the enhancement of the signal in the presence of the target DNA is not due to a spectral overlap. We detected DNA1 in the absence of DNA2 and DNA3. In this case, we observed the enhancement of the fluorescence signal for DNA1. No fluorescence enhancement was observed for DNA2 and DNA3 (Fig. S8a). We also detected DNA1 and DNA3 in the absence of DNA2 (Fig. S8b). Here, we observed the fluorescence enhancement for DNA1 and DNA3. No fluorescence enhancement was observed for DNA2. These experiments prove that the enhancement of the signal is not due a spectral overlapping.

In conclusion, we developed a novel DNA sensitive system that uses fluorescence quenching and DNA hybridization for detecting multiple DNA fragments simultaneously in a sample without separating them. This system is so specific that it can detect one mismatch in the sequence. This method yields consistent results and the time required for one assay is less than 5 minutes. This novel multiplex DNA detection system might serve as an alternative to traditional culture-based laboratory methods. In addition, amplification of pathogenic DNA is not required and the results are not obscured by the presence of proteins in the sample.

## Methods

### Assay procedure

We have designed the complementary DNA as per suspected target DNA in the sample. The complementary DNA fragments (C_DNA) were labeled at the 3′ position with selectively chosen fluorescence dye molecule. We have made a mixture of labeled complementary DNA fragments and LM (25 nM of labeled C_DNA1 + 100 nM of labeled C_DNA2 + 300 nM of labeled C_DNA3 + 30 µL of LM). This mixture had very low fluorescence as like background fluorescence. This mixture was added into the 1 mL solution of target DNA and recorded the fluorescence. The enhancement of fluorescence at the characteristic wavelengths corresponding to the respective fluorescence dye molecules indicates the presence of the suspected target DNA fragments. In this procedure, we can detect target DNA fragments up to concentration of 200 pM in a sample.

### Fluorescence quenching assay using LM

We have taken labeled C_DNA1 (25 nM), C_DNA2 (100 nM) and C_DNA3 (300 nM) in a micro tube, added 0.5 mM tris buffer (pH 7.5) and fluorescence was measured. Similarly, we have taken the same mixture of labeled complementary DNA in different micro tubes and added different volumes (2 µL, 5 µL, 10 µL, 15 µL, 20 µL, 30 µL) of LM solution from the stock solution of self-assembled LM (1 mg/mL). The fluorescence was recorded.

### DNA detection assay

We have taken labeled C_DNA1 (25 nM), C_DNA2 (100 nM) and C_DNA3 (300 nM) in a micro tube, added 0.5 mM tris buffer (pH 7.5) and fluorescence was measured (Solution-1). We have taken the same mixture of labeled complementary DNA fragments, added 30 µL of the self-assembled LM and recorded its fluorescence in the same buffer solution (Solution-2). Similarly, we have taken the same mixture of labeled complementary DNA, added 30 µL of LM and this mixture was added into the target DNA solutions, containing T_DNA1, T_DNA2, T_DNA3, having concentration 0.5 µM, 1 µM and 2 µM. We have recorded the fluorescence.

### Detection of DNA1 in absence of DNA2 and DNA3

The experimental procedure is same as described above in DNA detection assay. Here, we have added the solution-2 (mixture of labeled complementary DNA samples and 30 µL of LM) into the target DNA solution which contains only 1 µM of T_DNA1. We have recorded the fluorescence.

### Detection of DNA1 and DNA3 in absence of DNA2

The experimental procedure is same as described above in DNA detection assay. Here, we have added the solution-2 (mixture of labeled complementary DNA samples and 30 µL of LM) into the target DNA solution which contains 1 µM of each T_DNA1 and T_DNA3. We have recorded the fluorescence.

### DNA detection in presence of protein solution

We have taken labeled C_DNA1 (25 nM), C_DNA2 (100 nM) and C_DNA3 (300 nM) in a micro tube, added 30 µL of the self-assembled LM and recorded its fluorescence (Solution-2). Similarly, we have taken the same mixture of labeled complementary DNA, added 30 µL of LM and this mixture was added into the target DNA solutions having concentration 2 µM in absence and presence of BSA (1 mg/mL). We have recorded the fluorescence. We have performed similar experimental procedure with skim milk (1 mg/mL) instead of BSA.

### Identification of mismatched DNA

We have taken labeled C_DNA1 (25 nM), C_DNA2 (100 nM) and C_DNA3 (300 nM) in a micro tube, added 0.5 mM tris buffer (pH 7.5) and fluorescence was measured (Solution-1). We have taken the same mixture of labeled complementary DNA fragments, added 30 µL of the self-assembled LM and recorded its fluorescence in the same buffer solution (Solution-2). Similarly, we have taken the same mixture of labeled complementary DNA, added 30 µL of LM and this mixture was added into the target DNA solutions and mismatched DNA solution having concentration 2 µM. We have recorded the fluorescence.

### Identification of detection limit

We have taken labeled C_DNA1 (25 nM), C_DNA2 (100 nM) and C_DNA3 (300 nM) in a micro tube, added 30 µL of the self-assembled LM and recorded its fluorescence (Solution-2). Similarly, we have taken the same mixture of labeled complementary DNA, added 30 µL of LM and this mixture was added into the target DNA solutions having concentration 5 nM, 3 nM, 1.5 nM, 1 nM, 0.5 nM, 0.3 nM, 0.2 nM and 0.1 nM. We have recorded the fluorescence.

### Calibration curve

The calibration curve was drawn using the Origin software.

## Supplementary information

Supporting information
