# Supplementary material for: Multiplex optical detection and quantification of DNA fragments by metallo-peptide assemblies
Source: Sci Rep. 2019 Jun 19;9:8789. doi: 10.1038/s41598-019-45124-z (PMC6584589; doi:10.1038/s41598-019-45124-z)
Supplement: Supplementary file 1 — Supporting information [file 41598_2019_45124_MOESM1_ESM.docx]

**Electronic Supplementary Information**

**Multiplex optical detection and quantification of DNA fragments by metallo-peptide assemblies**

Abhijit Saha ^a,b^ and Meital Reches ^a,b^*

^a^ Institute of Chemistry The Hebrew University of Jerusalem, Edmond J. Safra Campus, Givat Ram, Jerusalem 9190401, Israel

^b^ The centre for Nanoscience and Nanotechnology. The Hebrew University of Jerusalem, Jerusalem 9190401, Israel

Email: meital.reches@mail.huji.ac.il.

**Materials and Methods**

**Materials:** All the target DNA fragments, the labeled complementary DNA fragments, mismatched DNA fragment, bovine serum albumin and tris base were purchased from the Sigma Aldrich. 1, 1, 1, 3, 3, 3-Hexafluoro-2-propanol (HFIP) was purchased from Merck.

**Instruments:** The absorbance of the labeled DNA solution was measured in UV-VISIBLE Spectrophotometer from Shimadzu. We have measured the fluorescence in the PerkinElmer Fluorescence Spectrometer LS55.

**Preparation of stock solution of DNA fragments:** We have prepared a 100 µM of stock solution of the DNA fragments by dissolving them into the 10 mM tris buffer having pH 7.5 and kept in 4 °C refrigerator.

**Preparation of working solution of LM:** 10 µL of HFIP was added into the 1 mg of solid LM and HFIP was evaporated out by using N_2_ flow. Now 375 µL of milliQ water was added into the solid. It was vortex and sonicated to get a suspension. It was kept for few hours at room temperature and lyophilized. The solid obtained was the self-assembled LM. It was dissolved into 1 mL of milliQ water and kept in 4 °C refrigerator for further use. The concentration of the solution is 1 mg/mL.

**Measurement of absorbance and fluorescence:** We have measured the absorbance and fluorescence of the labeled DNA fragments in 0.5 mM tris buffer at pH 7.5 at room temperature.

**Supplementary Figures:**

**Fig. S1** Principal compound for DNA detection system. This is the ligand metal conjugate (LM) or metallo-peptide. The self-assembled structure of this compound has been used for DNA detection.


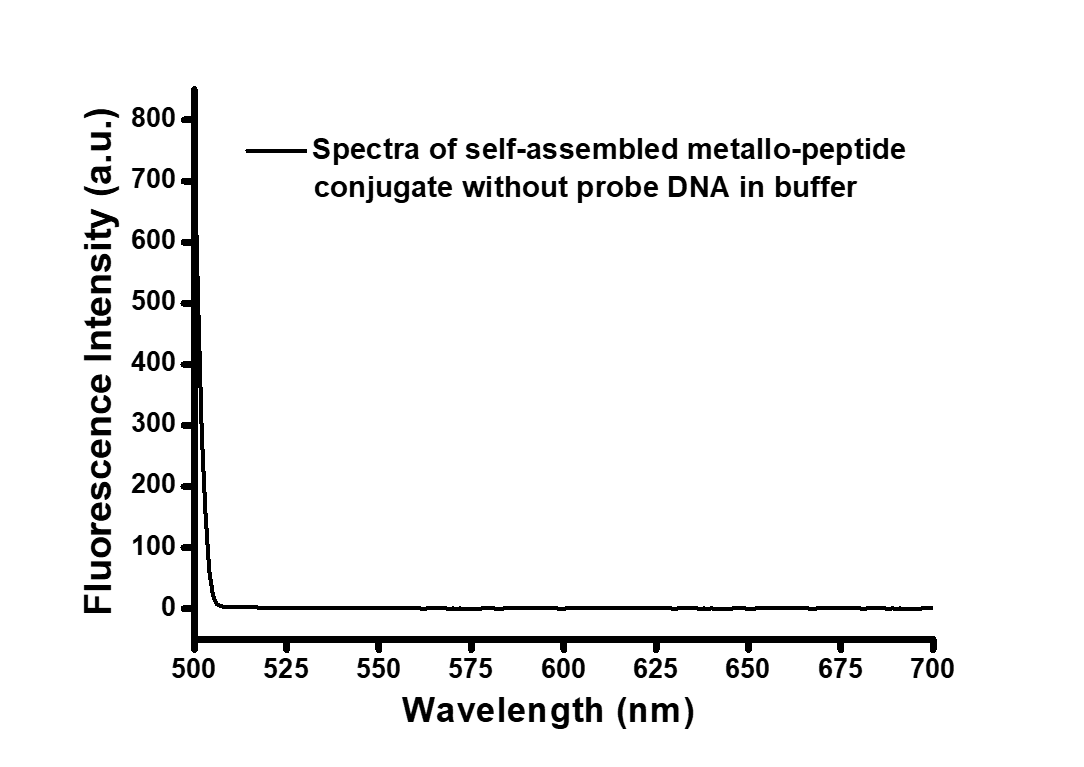


**Fig. S2** Fluorescence spectra of the self-assembled metal-peptide conjugate (LM) without probe DNA (labelled complementary DNA) using the excitation wavelength at 493 nm. It is the control spectra showing no fluorescence peaks in the selected range of wavelength for our DNA detection.

**Table 1a**

| **Meningitis Pathogens** |  | **Target DNA sequence (5´- 3´)** |
| --- | --- | --- |
| Streptococcus pneumoniae | DNA 1 | TTCGAGTGTTGCTTATGGGCGCCA **(T_DNA1)** |
| Haemophilus influenzae | DNA 2 | CCACGCTCATTCGTTTGATGAGTGGTG **(T_DNA2)** |
| Neisseria meningitidis | DNA 3 | ATGTGCAGCTGACACGTGGCAATG **(T_DNA3)** |
| Mismatched DNA | DNA 1 (MM) | TTCGAGTGTTGCTTATGTGCGCCA **(T_DNA1_MM)** |

**Table 1b**

| **Complementary DNA or Probe DNA sequence (5´- 3´)** | **Modification at 3´ positions** |
| --- | --- |
| TGGCGCCCATAAGCAACACTCGAA (C_DNA1) | Alexa Fluor 488 (Em: 525 nm) |
| CACCACTCATCAAACGAATGAGCGTGG (C_DNA2) | Cyanine 3 (Em: 568 nm) |
| CATTGCCACGTGTCAGCTGCACAT (C_DNA3) | Texas Red (Em: 615 nm) |

**Table S1** Description of the target DNA sequence of meningitis pathogens, mismatched DNA and the complementary DNA sequence corresponding to the target DNA sequence.

**
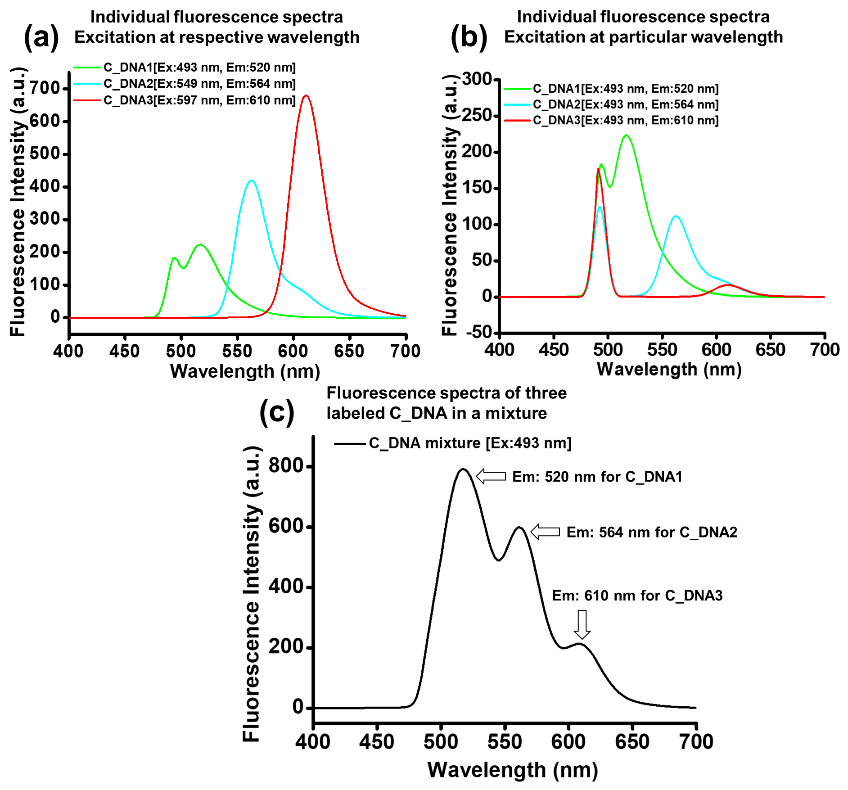
**

**Figure S3.** (a) Individual fluorescence spectra of the labelled DNA samples using their respective excitation wavelengths. (b) Individual fluorescence spectra of the A488, Cy3 and TxRd labelled DNA samples using excitation at 493 nm. (c) Fluorescence spectrum of the three labelled DNA samples in a mixture using excitation at 493 nm.


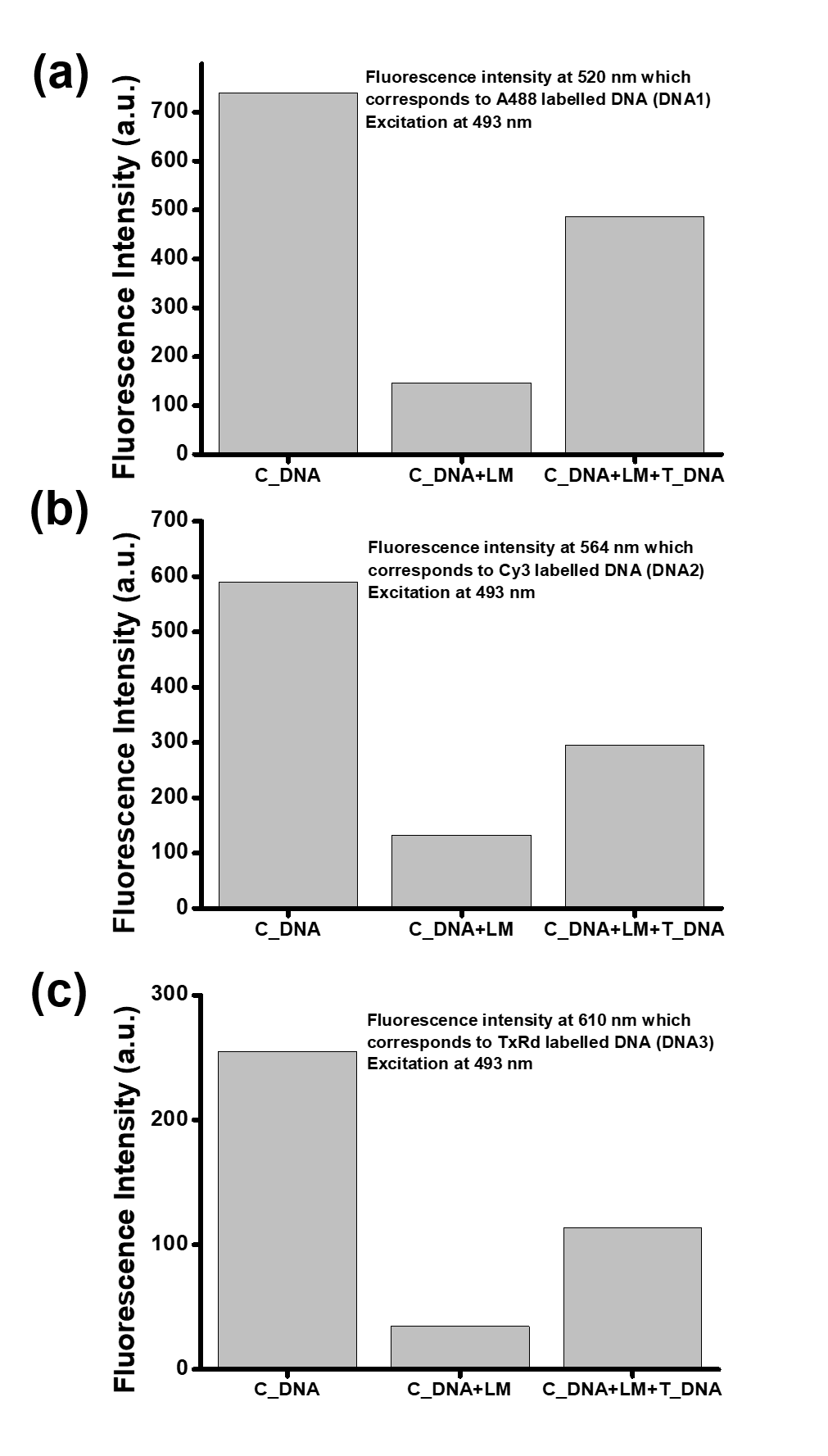


**Fig. S4** Bar diagrams indicate the fluorescence quenching by LM and recovery of fluorescence on addition of target DNA.

**Fig. S5** Detection of three DNA samples in the presence of skim milk, indicating the selectivity of the system.

**Fig. S6** Bar diagram indicates that the enhancement of all three fluorescence simultaneously on addition of 0.2 nM (200 pM) of target DNA which indicating lower limit of DNA detection.

**Fig. S7** Calibration curve for simultaneous detection of three DNA at very low concentration. Enhancement of fluorescence signals are decreasing with lowering the concentration of target DNA. The spectrum corresponding to 0.1 nM T_DNA was overlapped on the spectrum of (C_DNA mixture + LM).


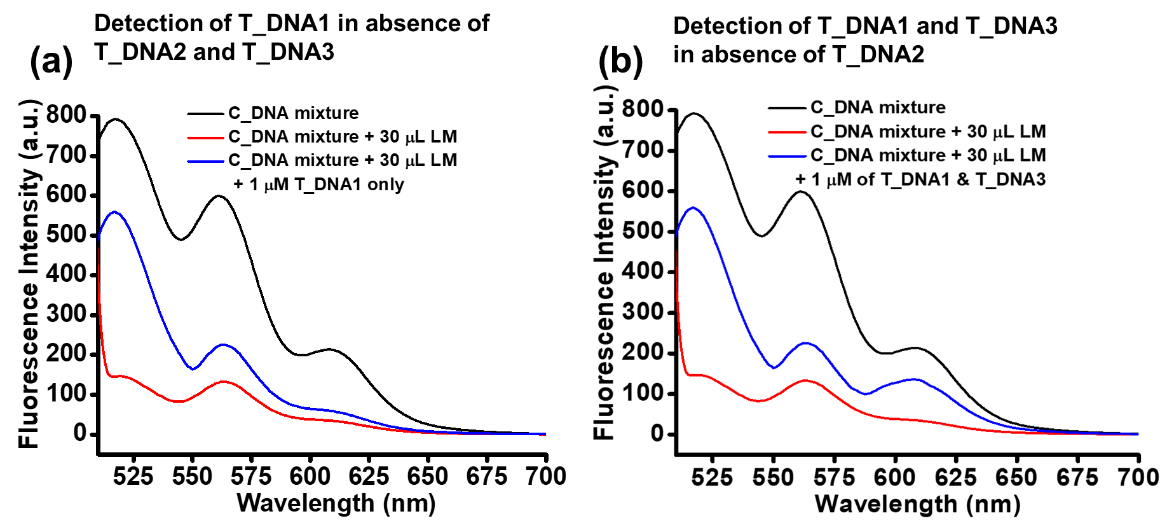


**Fig. S8** Control experiment showing detection of particular DNA samples in absence of other DNA samples.
